# 基于微流控技术的磁免疫荧光法在EB病毒检测中的应用

**DOI:** 10.3724/SP.J.1123.2021.09005

**Published:** 2022-04-08

**Authors:** Junhao LI, Guanhua HAN, Xiaotao LIN, Liqiang WU, Chungen QIAN, Junfa XU

**Affiliations:** 1.广东医科大学医学技术学院检验医学研究所临床免疫学教研室, 广东 东莞 523808; 1. Department of Clinical Immunology, Institute of Laboratory Medicine, Medical Technology College, Guangdong Medical University, Dongguan 523808, China; 2.深圳市亚辉龙生物科技股份有限公司, 广东 深圳 518100; 2. Shenzhen YHLO Biotechnology Company Limited, Shenzhen 518100, China

**Keywords:** 微流控, 磁免疫荧光, EB病毒, 快速检测, microfluidic, magnetic immunofluorescence, Epstein-Barr virus (EBV), rapid detection

## Abstract

EB病毒(Epstein-Barr virus, EBV)的早期诊断能够降低患者发生重大疾病的风险。临床上常用的EBV抗体的检测方法存在耗时长、试剂消耗大和效率低等缺点。相比于传统的检测方法,微流控(microfluidics)技术具有高通量、试剂消耗少,污染少和自动化程度高等优点,磁免疫荧光技术具有检测效率高、信号强等优点,将两者的优势结合,能够弥补传统方法的不足。鉴于此,采用聚甲基丙烯酸甲酯(PMMA)作为芯片原材料,经过激光切割及真空热压加工工艺能够快速获得芯片。将包被抗原的磁珠及包被抗人抗体的荧光微球经过冷冻干燥工艺快速冻干成小球并嵌入芯片内,经过孵育和清洗后,进行检测。通过图像分析快速得到检测结果。通过精密度、特异性、剂量-反应曲线及检出限测试,进行性能验证。通过与化学发光免疫分析法(CLIA)检测的临床样本比对,进行方法学与临床应用评价。结果显示相对标准偏差(RSD)均小于10%。与多种常见的病原体抗体均无交叉反应。EB病毒衣壳抗原(Epstein-Barr viral capsid antigen, EB VCA)IgG项目的检出限为1.92 U/mL,线性范围为1.92~200 U/mL,阳性符合率为95.77%(68/71),阴性符合率为86%(43/50); EB VCA IgA项目的检出限为2.79 U/mL,线性范围为2.79~200 U/mL,阳性符合率为92%(46/50),阴性符合率为92.96%(66/71); EB病毒核心抗原1(Epstein-Barr nuclear antigen 1, EB NA1)IgG项目的检出限为3.13 U/mL,线性范围为3.13~200 U/mL,阳性符合率为92.96%(66/71),阴性符合率为92%(46/50); EB NA1 IgA项目的检出限为1.53 U/mL,线性范围为1.53~200 U/mL,阳性符合率为90%(45/50),阴性符合率为91.55%(65/71)。4个项目能在20 min内快速完成检测,且与临床上使用CLIA方法测试的结果具有良好的相关性,可以为临床提供一种快速、灵敏、简便、自动化程度高和易于基层推广的检测方法。

## Abstract (en)

Early diagnosis of Epstein-Barr virus (EBV) can reduce the risk of major illnesses. Disadvantages of EBV antibody detection methods that are commonly used clinically include lengthy assay time, need for a lot of reagent, and low efficiency. Compared with traditional detection methods, microfluidics technology offers high throughput, low reagent consumption, less bio-contamination, and a higher degree of automation. Advantages of magnetic immunofluorescence technology include high detection efficiency and a strong signal. The combined advantages of the two methods can compensate for the shortcomings of traditional methods. In the present study, polymethyl methacrylate (PMMA) as the raw material was subjected to laser cutting and vacuum hot pressing to quickly obtain chips. Magnetic beads labeled with antigen and fluorescent microspheres labeled with anti-human antibody were then rapidly lyophilized into microspheres by freeze-drying and embedded into the chips. After incubation and cleaning, the last step was detection. Image J software was used to analyze the mean fluorescence intensity and obtain negative or positive test results. To determine the precision of the chip, high- and low-value samples of each item were retested 10 times. The mean values were calculated to obtain the relative standard deviation (RSD) for several common pathogens. Furthermore, the coincidence rate of clinical samples was tested using a chemiluminescence immunoassay (CLIA) to determine the potential clinical application value. The RSD of the precision test for each item was <10%, indicating good precision. The precision of the accelerated stability test was not verified. Specificity test results revealed no cross-reaction with some common pathogen antibodies, indicating good specificity. It remains to be verified whether the antibodies detected by this method cross-react with other herpes simplex viruses, such as types 1 and 2, Kaposi’s sarcoma-associated virus, and human herpes virus type 6 and 7. Of the 121 clinical samples tested, statistical analysis of the data indicated good agreement with the chemiluminescence immunoassay in clinical trials. EB viral capsid antigen (EB VCA) IgG positive coincidence rate was 95.77% (68/71), the negative coincidence rate was 86% (43/50) (Kappa=0.828, *P*<0.05), the limit of detection (LOD) was 1.92 U/mL, and the linear range was 1.92 to 200 U/mL. The EB VCA IgA positive coincidence rate was 92% (46/50), negative coincidence rate was 92.96% (66/71) (Kappa=0.847, *P*<0.05), LOD was 2.79 U/mL, and the linear range was 2.79 to 200 U/mL. The positive coincidence rate of EB nuclear antigen 1 (EB NA1) IgG was 92.96% (66/71), the negative coincidence rate was 92% (46/50) (Kappa=0.847, *P*<0.05), the LOD was 3.13 U/mL, and the linear range was 3.13 to 200 U/mL. The positive coincidence rate of EB NA1 IgA was 90% (45/50), the negative coincidence rate was 91.55% (65/71) (Kappa=0.813, *P*<0.05), the LOD was 1.53 U/mL , and the linear range was 1.53 to 200 U/mL. Compared with the traditional enzyme-linked immunosorbent assay, the novel method featured a shorter detection time, reduced use of reagent, high degree of automation, and less bio-contamination. Compared with CLIA, advantages of the novel method include multi-item combined detection, long luminescence time, and simple use as a basic health service. Compared with silicon and ceramic microfluidic chips, advantages of the selected PMMA material include low processing cost, short processing time, simple processing technology, and easy industrialization. A refinement that can still be made include the use of molding instead of laser cutting technology, which can further shorten the chip processing time. In summary, a microfluidic detection platform was initially built to provide a rapid, sensitive, simple, highly automated, and easy to be used by basic health service for the quantitative combined detection of EBV VCA and EB NA1 IgG and IgA.

EB病毒(Epstein-Barr virus, EBV)是一种*γ*-疱疹病毒亚科的病毒。EBV感染与多种疾病有关,从急、慢性炎症到恶性肿瘤,EBV都被认为参与了这些疾病的发病机制^[1]^。在EBV相关的恶性肿瘤中,鼻咽癌和胃癌是EBV相关死亡的最常见的原因^[2,3,4,5,6,7,8]^。由于患者的个体差异,单个抗体的检测容易漏检、误检。多个EBV抗体联合检测,可以为临床提供更准确的诊断依据。例如:Epstein-Barr viral capsid antigen (EB VCA) IgA和EB病毒核心抗原1(EB NA1) IgA是最常用的鼻咽癌筛查标志物,EB VCA IgG、EB NA1 IgG的检测结果有助于判断患者是否属于既往感染^[9,10,11]^。用ELISA的方法单独检测EB VCA IgA和EB NA1 IgA,特异性分别为83.8%和80.0%,而联合检测EB VCA IgA和EB NA1 IgA,特异性则高达90.6%。因此,EB VCA IgG、EB VCA IgA、EB NA1 IgG及EB NA1 IgA四者联合检测可以提高检测的特异性及准确性,为临床诊断决策提供更加全面且完整的检测依据^[12,13,14,15,16]^。

目前临床上检测EBV抗体的方法主要为ELISA法和化学发光免疫分析法(CLIA)法^[17,18,19]^。传统的ELISA方法存在检测时间较长,试剂用量大,自动化程度不高等缺点,而CLIA法也存在仪器过大,发光时间短,不适合在基层医疗机构推广等缺点。近年来,在医学科学与高新技术的碰撞下,具有检测操作简单化、报告结果即时化等优点的即时检验(point of care testing, POCT)越来越受到人们的青睐。微流控技术(microfluidics)的优点在于能够对流体体积及流速进行精确的控制。在检测项目时,能够保证高精度及灵敏度。同时还具有试剂及样本消耗少,高通量,设备小型化及自动化等优点。当新的传染病暴发流行时,能够快速、简便地获取检测结果^[20,21,22,23]^。因此,微流控技术在POCT领域具有十分广阔的应用前景^[24,25,26,27,28]^。Gao等^[29]^基于微流控技术建立了前列腺特异性抗原肿瘤标志物快速定量检测方法,其线性范围可以达到0.05~200 ng/mL。Ng等^[30]^也基于微流控技术建立了促甲状腺激素和17*β*-雌二醇检测的微流控芯片,每个项目在6 min内即可出结果,而传统的ELISA方法则至少需要60 min。

目前,市场上已有很多商业化的微流控芯片。其中,做免疫检测微流控芯片较为成熟的厂家主要有瑞典Gyros公司及葡萄牙Biosurfit公司等。其中,Gyros公司设计的Gyros^TM^微流控免疫测定平台主要用于药代动力学及免疫原性分析^[31]^,其芯片由于加工精度的要求,成本较高。Biosurfit公司利用了表面等离子共振原理设计的Spinit微流控平台,在免疫检测上主要用于C-反应蛋白的检测^[32]^。其芯片结构相对复杂,芯片加工成本较高且仪器成本也较高。鉴于此,我们基于微流控技术及磁微粒免疫荧光分析技术专门设计出联合检测EB VCA IgG、EB VCA IgA、EB NA1 IgG及EB NA1 IgA的微流控芯片,在芯片设计上摒弃了很多造价高的设计,但不影响试剂在芯片内的性能,可以降低并控制成本及芯片结构的稳定性(批间差)。该芯片兼具小型化与自动化、检测试剂消耗少、污染少、检测快速等优点。

## 1 实验部分

### 1.1 仪器、试剂与材料

VLS3.50激光切割机(美国Universal Laser Systems公司); TBS-200微流控芯片真空热压机(浙江扬清芯片技术有限公司); Sorvall Legend Micro 21R高速微量离心机(美国Thermo公司);超声波细胞粉碎机(宁波新芝生物公司); Epsilon 2-4 LSCplus真空冷冻干燥机(德国Martin Christ公司);涡旋振荡器(美国Labnet公司); DCM8激光共聚焦显微系统(德国Leica公司);微流控芯片检测样机、iFlash3000化学发光免疫分析仪(深圳市亚辉龙生物科技股份有限公司)。

抗人IgA单克隆抗体(5 mg/mL)和抗人IgG单克隆抗体(5 mg/mL)购自美国Sigma-Aldrich公司;羧基磁珠(10 mg/mL)和羧基荧光微球(10 mg/mL)购自美国Thermo公司;EB VCA抗原(5 mg/mL)和EB NA1抗原(5 mg/mL)购自深圳市亚辉龙生物科技股份有限公司;牛血清白蛋白(BSA)(分析纯,上海阿拉丁生化科技股份有限公司);海藻糖、ProClin300、NaCl、*N*-羟基琥珀酰亚胺(NHS)、1-(3-二甲氨基丙基)-3-乙基碳二亚胺盐酸盐(EDC)(分析纯,美国Sigma-Aldrich公司);聚甲基丙烯酸甲酯(PMMA)板材(成都世化亚克力科技有限公司);压敏胶(美国3M公司)。2-(*N*-吗啉代)乙磺酸(MES)、2-(4-(2-羟乙基)哌嗪)乙磺酸(HEPES)、磷酸二氢钾、磷酸氢二钠(分析纯,上海国药集团化学试剂有限公司);磷酸盐缓冲液(PBS)粉末(10 mmol/L)购自北京利维宁生物科技有限公司;液氮购自深圳昌达利气体服务中心。

### 1.2 微流控芯片制备方法

本芯片采用激光切割技术及真空热压相结合的加工工艺,具体步骤为:首先绘制芯片各层二维结构图,如图1。然后进行各层三维结构设计及优化,最终得到如图2a所示共5层的三维结构示意图,分别为顶层、腔室存储层、流体通道层、柔性阀门层、交互控制层。将平面结构图导入VLS3.50 Universal激光切割机的配套软件里,以PMMA板材为芯片材料,按照图1所示,切割出如图2a的5层结构,并按照图2a顺序贴好,放入微流控真空热压机下,在背压0.5 MPa、正压0.7 MPa, 50 ℃的条件下热压5 min。即可得到如图3a所示的微流控芯片。材料及贴胶要求见图1注解,阀控结构见图2c及2d。

**图1:**
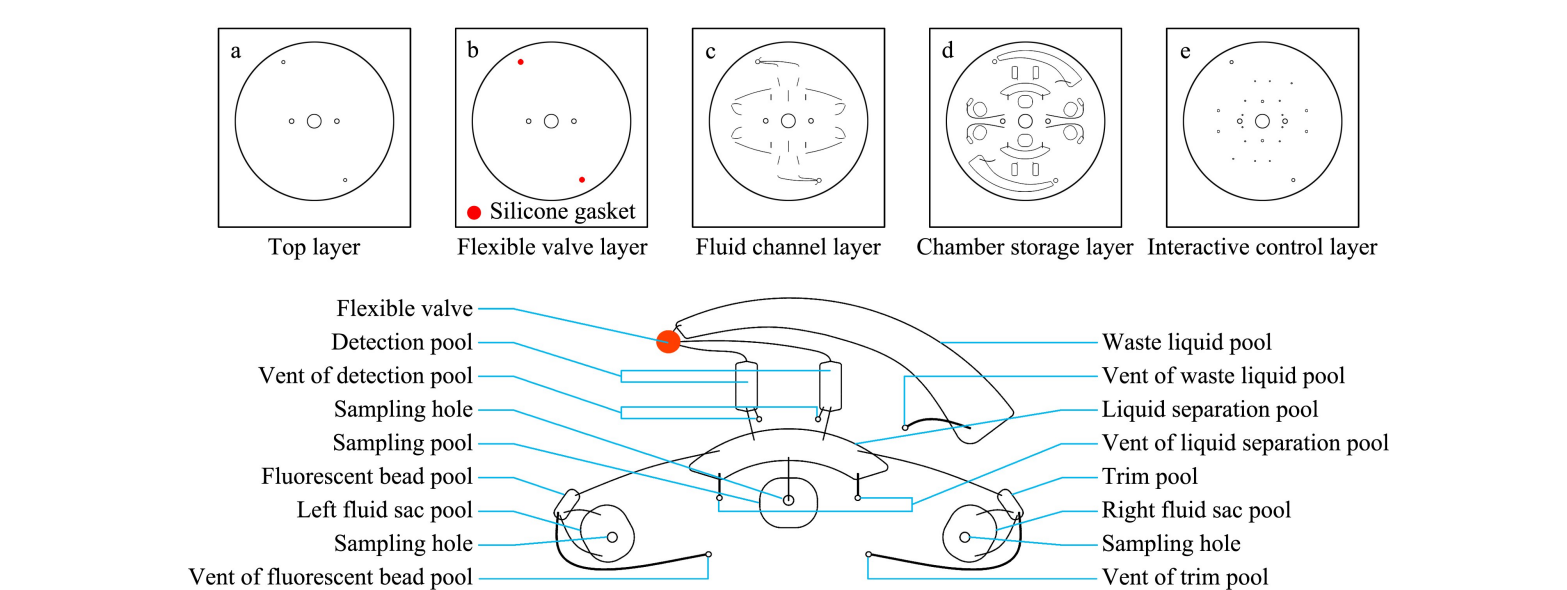
芯片二维结构示意图

**图2:**
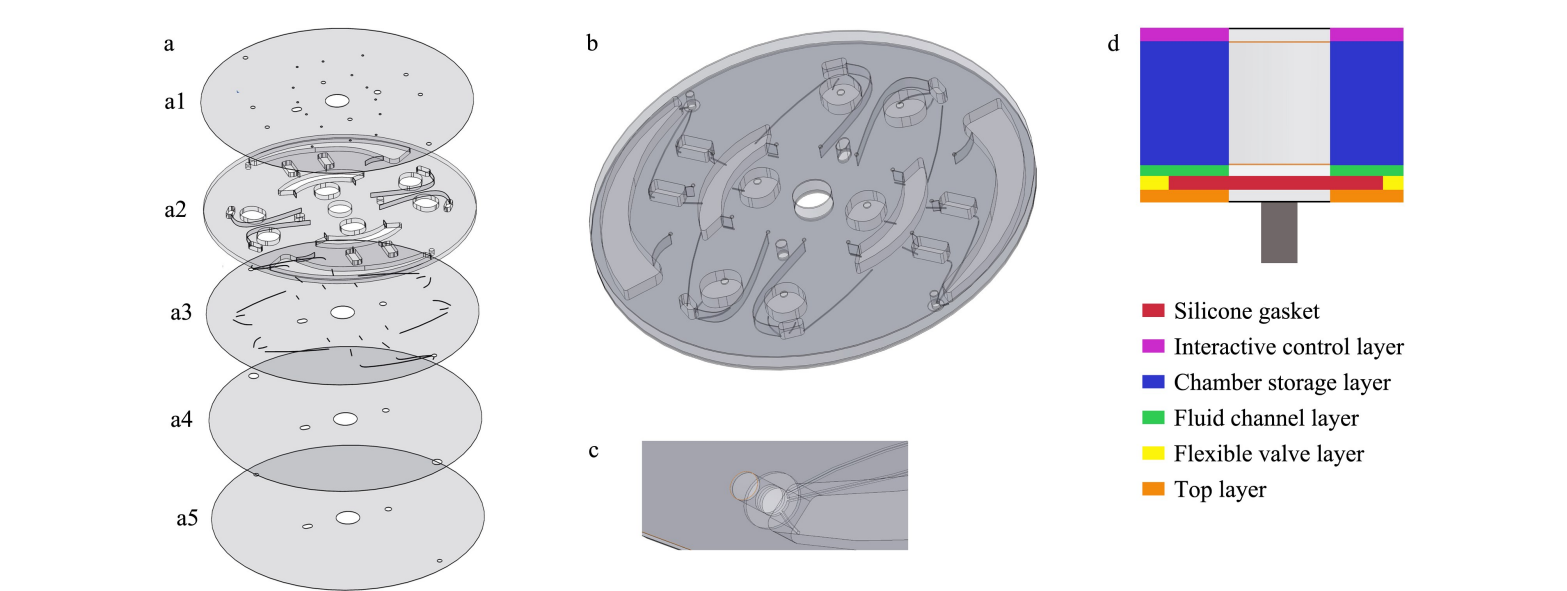
芯片的三维结构示意图

**图3:**
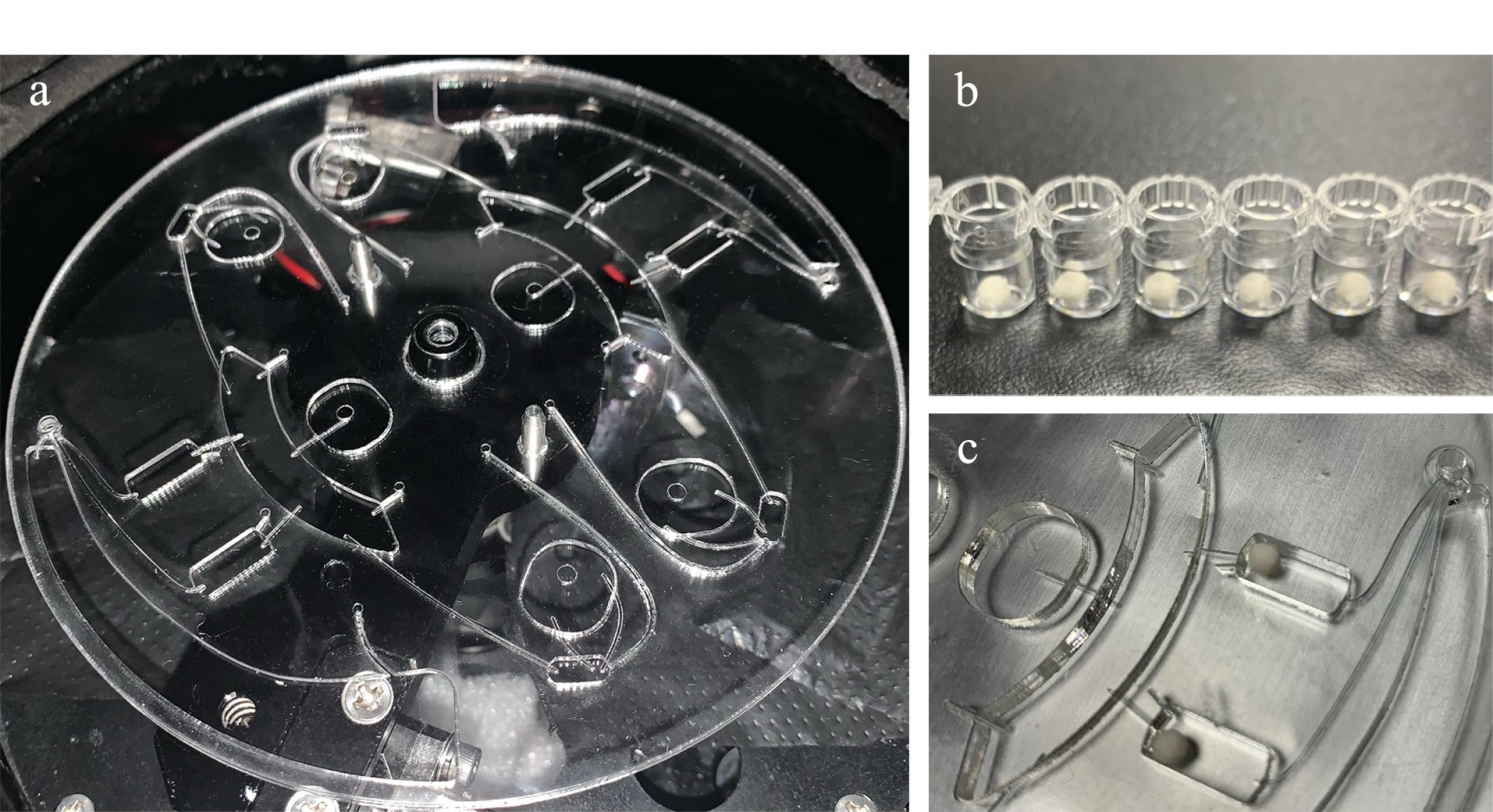
芯片及冻干微球实物图

### 1.3 微流控芯片试剂制备方法

1.3.1 EB VCA、EB NA1抗原偶联羧基磁珠方法

以制备2 mL 5 mg/mL的EB VCA抗原偶联磁珠为例,EB NA1抗原偶联磁珠同理。方法:首先移取0.5 mL(10 mg)的1 μm的羧基磁珠,用5 mL的MES (50 mmol/L, pH 6.0)缓冲液洗涤3次。然后加入活化试剂EDC和NHS各25 mg,在室温下,将离心管置于旋转混匀仪上活化反应30 min。再将200 μL的EB VCA抗原加至5 mL的MES(50 mmol/L, pH 6.0)缓冲液中,配制成抗原液,将抗原液加到装有活化磁珠的离心管内,在室温下,将离心管置于旋转混匀仪上偶联反应24 h。弃去上清液后再洗涤3次。最后一次洗涤后,重悬磁珠于2 mL洗涤缓冲液内,此时磁珠的质量浓度约为5 mg/mL。

1.3.2 抗人IgA、抗人IgG偶联羧基荧光微球方法

以制备1 mL 0.5 mg/mL 抗人IgG偶联的荧光微球为例,抗人IgA偶联荧光微球的方法同理。方法:首先取10 mg羧基微球,加入1 mL MES(50 mmol/L, pH 6.0)缓冲液,在14000 r/min的条件下离心10 min。弃去上清液,加入800 μL的MES(50 mmol/L, pH 6.0)缓冲液,进行超声。然后称取10 mg NHS和10 mg EDC分别溶于1 mL和2.5 mL的MES(50 mmol/L, pH 6.0)缓冲液中,振荡混匀后,分别将100 μL NHS溶液和EDC溶液滴加到制备好的荧光微球溶液中,振荡混匀。室温下,避光旋转混匀20 min。离心后弃去上清液。加入0.8 mL HEPES缓冲液(20 mmol/L, pH 7.4)到离心管中,超声1 min。然后取100 μL 抗人IgG(5 mg/mL)抗体溶液加入到离心管中,振荡混匀,室温下避光旋转混匀2 h。然后加入100 μL BSA溶液(20 mg/mL)封闭。室温下,避光旋转混匀1 h。离心后弃去上清液。加入1 mL PBS溶液(50 mmol/L, pH 7.4, 含0.2%(质量分数) Tween 20+5%(质量分数)海藻糖),超声后即可得到0.5 mg/mL鼠抗人IgG偶联的荧光微球溶液1 mL。

1.3.3 荧光微球及磁珠冻干方法

首先配制缓冲液,以1 L纯水配制为例:向1 L纯水中加入1.96 g PBS粉末、8.77 g NaCl、10 g BSA、10 g海藻糖、1 g ProClin300。用足量的缓冲液按照100:1及10:1的比例分别将偶联好抗原的磁珠母液、偶联好抗体的荧光微球母液稀释成工作液。将1 L液氮倒入保温盒中,再将酶标板放入保温盒,盛满液氮。用移液枪移取工作液20 μL,垂直在酶标板孔的上方点液,工作液会在液氮的条件下速冻成球型。再将冻干小球转移到真空冻干机里,进行预冻、主干燥和终末干燥。将冻干后的小球放置在干燥环境下保存(见图3b及3c)。

### 1.4 微流控芯片检测仪器设计

仪器硬件及软件由深圳亚辉龙生物科技股份有限公司(YHLO)相关专业人员设计,硬件主要有离心模块、荧光显微系统、磁吸控制模块、自动化顶针模块及芯片固定模块等硬件。

### 1.5 微流控芯片反应原理及使用方法

采用磁免疫荧光法原理,形成磁珠-抗原-抗体-二抗-荧光微球复合物,见图4a。操作的详细方法见图4b、4c、4d、4e及表1,简述如下。

**图4:**
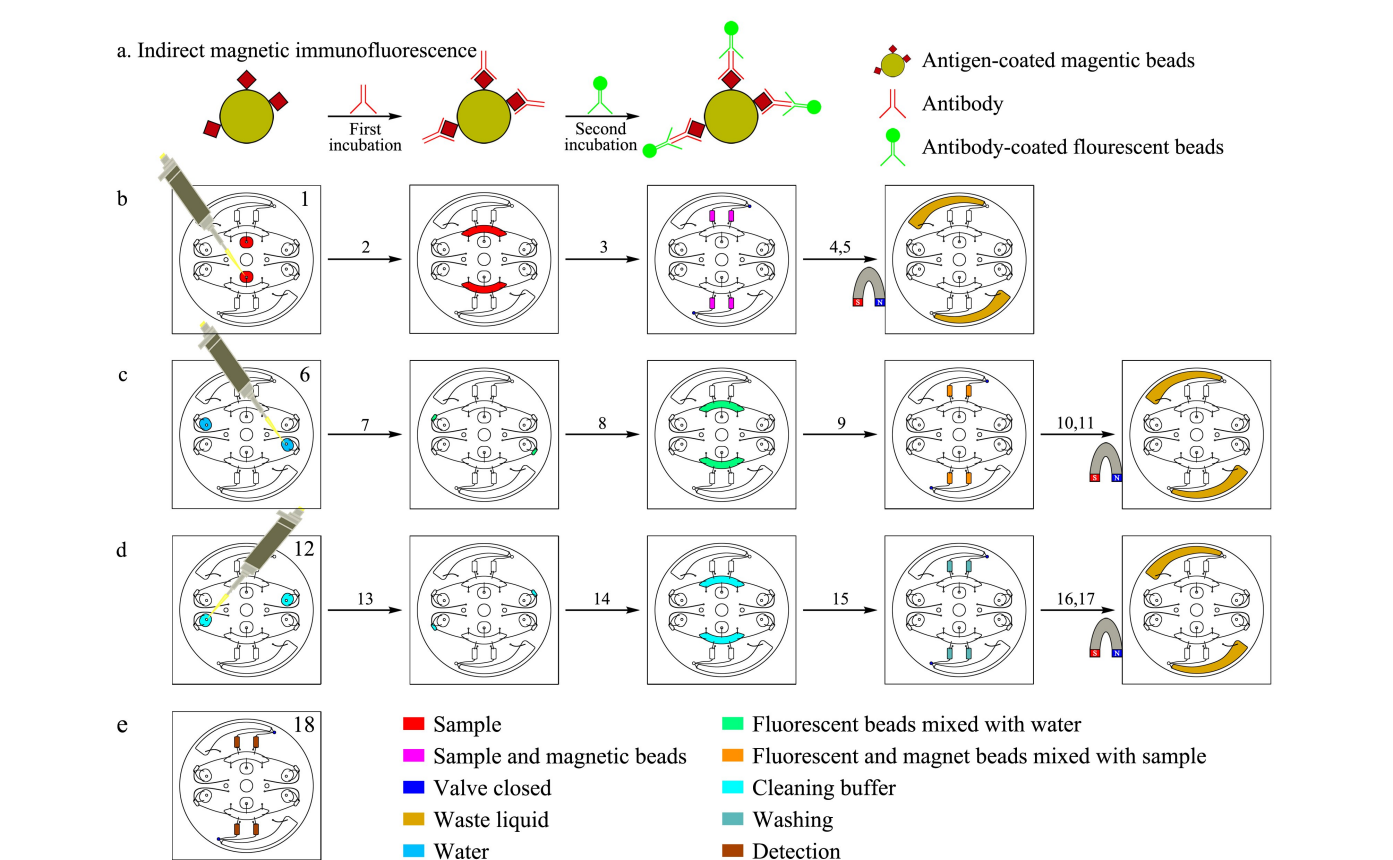
芯片检测流程示意图

**表1:** 芯片检测流程控制程序

Stage	Number in Fig. 4	Rotational speed/(r/min)	t/s	Control	Specification
First incubation	1	-	-	-	sampling
	2	500	10	valve close	sample guiding
	3	800	10	valve close	sample aliquoting
	4	500	300	valve close	reacting (bi-directional running with 120°)
	5	800	10	magnetic beads accumulation; valve open	draining
Second incubation	6	-	-	magnetic beads re-suspension; valve close	adding deionized water (DW)
	7	300	10	valve close	DW guiding
	8	500	10	valve close	DW guiding
	9	800	10	valve close	DW aliquoting
	10	500	300	valve close	reacting (bi-directional running with 120°)
	11	800	10	magnetic beads accumulation; valve open	draining
Cleaning	12	-	-	magnetic beads re-suspension; valve close	adding cleaning buffer (CB)
	13	300	10	valve close	CB guiding
	14	500	10	valve close	CB guiding
	15	800	10	valve close	CB aliquoting
	16	500	300	valve close	cleaning (bi-directional running with 120°)
	17	800	10	magnetic beads accumulation; valve open	draining
Detection	18	-	-	-	signal collecting

-: no control conditions.

第一步孵育:使用加样枪在样本腔的加样孔点样(样本),操作仪器配套软件,在给定的转速、时间、阀门及磁铁吸附状态条件下,6 min内自动完成第一步孵育。

第二步孵育:使用加样枪在荧光微球池的液囊腔加样孔加去离子水,在仪器控制下6 min内自动完成第二步孵育。

清洗:使用加样枪在清洗液池的液囊腔加样孔加清洗液,在仪器控制下6 min内自动完成清洗。

检测:通过便携式荧光显微镜获取荧光微球的图片,通过Image J图像分析软件将获取的荧光图片拆分成红、绿、蓝3个通道,用Image J对绿色通道图进行荧光信号识别,得到平均荧光强度。

### 1.6 标准品配制、最佳临界值确定方法

标准品配制方法:收集5例临床上采用YHLO化学发光免疫分析仪(iFlash3000)测定为阳性的高值血清样本,将这5例样本混合后,用iFlash3000再次检测3次,所检出的浓度取平均值作为样本的真实浓度。用样本稀释液将已知浓度的混合血清进行稀释。分别配制成0、6.25、15、25、50、200 U/mL共6个浓度的系列参考标准品。标准品每瓶1 mL分装,于4 ℃保存备用。

最佳临界值确定方法:以临床样本的iFlash3000测试结果(阴阳性判断)作为参考,对本方法测试结果的换算浓度使用OriginPro 2021软件进行受试者工作特征曲线(receiver operating characteristic curve, ROC curve)分析,确定本方法的最佳临界值(optimal operating point, OOP)。

进行其他样本测定时,高于最佳临界值则定义为阳性,低于最佳临界值则定义为阴性。

### 1.7 性能评价指标及统计学处理

剂量-反应曲线:以自制试剂标准品浓度为横坐标(*X*),以平均荧光强度为纵坐标(*Y*),采用Graphpad Prism 9.0软件拟合标准曲线方程,相关系数(*R*^2^)≥0.99, *P*<0.05表明线性良好。检出限:平行测定20次零浓度标准品的平均荧光强度,计算平均值(AV)及标准差(SD),以均值AV+2SD的反应量代入标准曲线方程,所对应的浓度则为本方法的检出限。

重复性:选取每个项目的高、低值质控品重复测试10次,统计测试均值并计算相对标准偏差(RSD)。

特异性:本实验室收集的巨细胞病毒(Cytomegalovirus, CMV)抗体IgG、弓形虫(Toxoplasma Gondii, Toxo)抗体IgG、肺炎支原体(Mycoplasma pneumoniae, MP)抗体IgG、风疹病毒(Rubella virus, RV)抗体IgG、水痘-带状疱疹病毒(Varicella-zoster virus, VZV)抗体IgG、EB VCA IgG、EB NA IgG、EB VCA IgA、EB NA IgA抗体阳性样本,用本微流控芯片进行交叉反应检测,分析芯片的特异性。

统计学处理:采用Graphpad Prism 9.0软件拟合标准曲线方程,对微流控芯片法与CLIA法检测结果的相关性进行验证,*R*^2^≥0.95,表明相关性良好。采用SPSS 26.0软件对微流控芯片法与CLIA方法检测结果进行Kappa检验,若Kappa值在0.8~1之间,*P*<0.05,则可以认为两种方法的检测结果一致性较好。采用OriginPro 2021软件制作Bland-Altman图对微流控芯片法与CLIA方法临床测试结果进行一致性分析,统计95%置信区间外的样本数量占比。

## 2 结果与讨论

### 2.1 芯片通道结构表征

利用共聚焦显微镜对芯片进行扫描获得芯片通道明场图(见图5a)和芯片通道三维结构图(见图5b),通过相关配套软件可以获得最大深度和通道横截面积图。深度图可以直观地反映出芯片通道加工好坏及最大纵深距离,而通道横截面积则是计算单位时间内流过通道的液体体积的重要参数,最大深度及横截面积数据见表2,各个通道的平均深度在90~100 μm之间,这是本研究所用的激光切割工艺在保证通道加工状态良好的情况下能达到的最小深度范围。

**图5:**
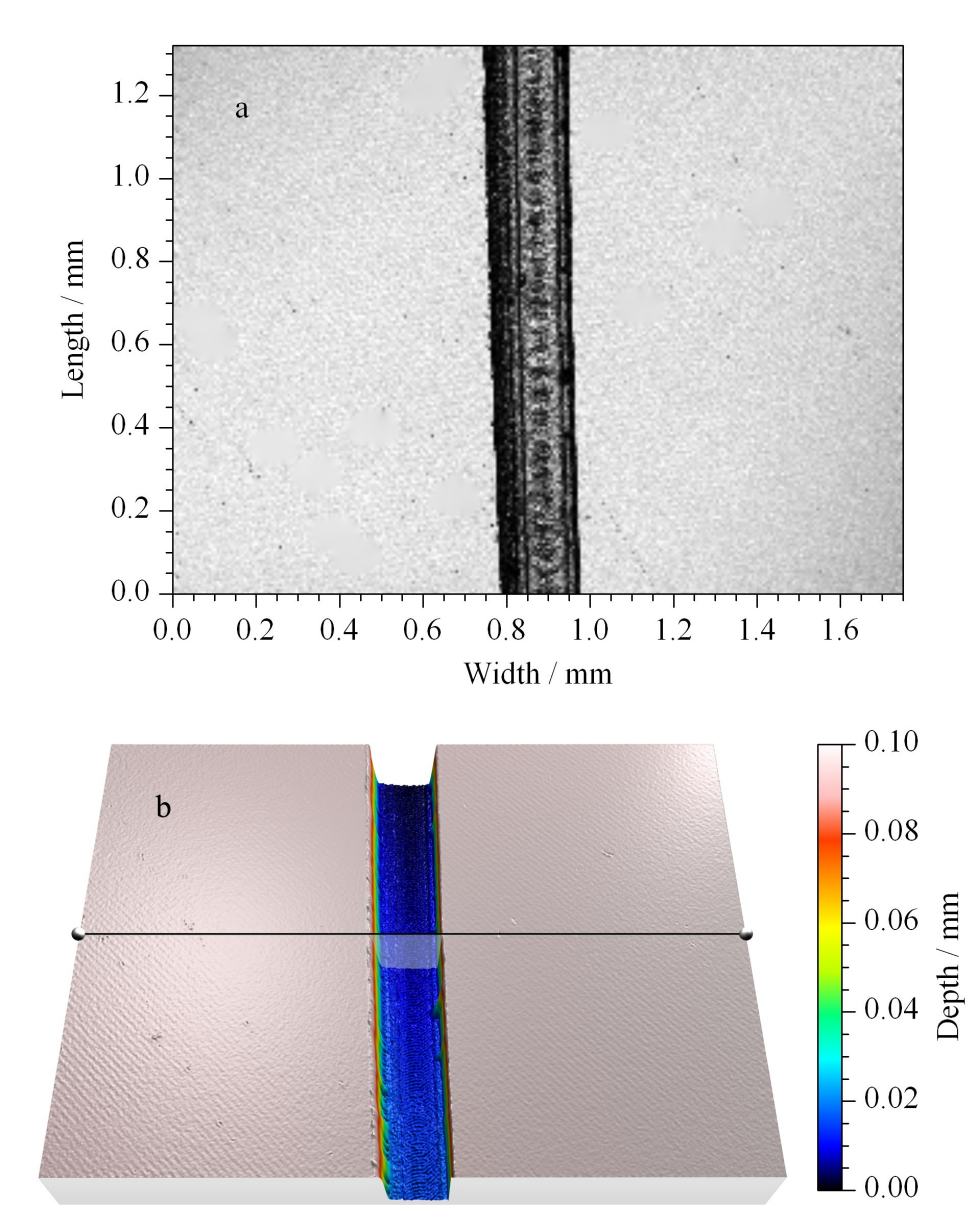
检测区在共聚焦显微镜下的(a)明场及(b)三维示意图

**表2:** 各通道最大深度及通道横截面积

Channel location	Maximum channel depth/μm	Channel cross-sectional area/μm^2^
Between sampling pool and liquid separation pool	93.51±2.94	15935.2±712.5
Between fluorescent bead pool and liquid separation pool	96.44±3.28	16718.7±654.6
Between left fluid sac pool and fluorescent bead pool	92.43±3.66	15502.3±857.4
Between detection pool and waste liquid pool	96.93±2.78	16614.1±811.3

### 2.2 最佳临界值

各项目经过ROC曲线测试,各项目ROC曲线见图6, OOP、曲线下方面积(area under curve, AUC)、OOP对应的敏感度及特异性见表3。

**图6:**
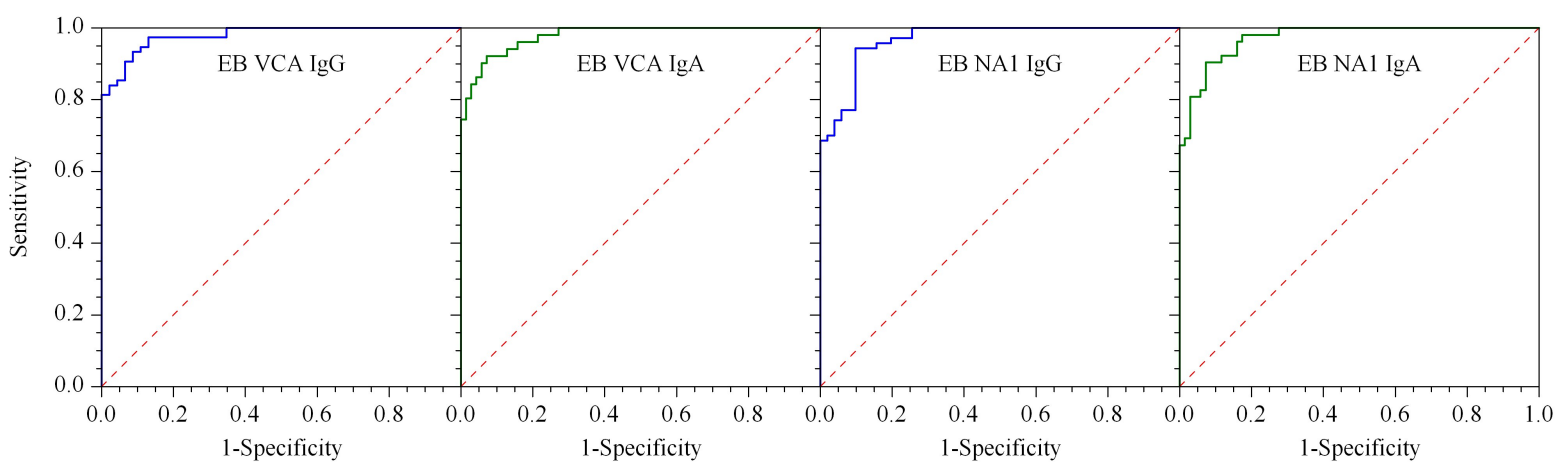
EB VCA IgG、EB VCA IgA、EB NA1 IgG及EB NA1 IgA的ROC曲线(*n*=121)

**表3:** ROC曲线下最佳临界点、灵敏度、特异性、曲线下方面积及参考样本数

Item	Normal serum sample number	Positive serum sample number	OOP/(U/mL)	Sensitivity/%	Specificity/%	AUC
EB VCA IgG	46	75	9.97	93.3	91.3	0.9788
EB VCA IgA	70	51	9.95	92.2	92.9	0.9784
EB NA1 IgG	51	70	9.97	94.3	90.2	0.9672
EB NA1 IgA	52	69	10.05	90.4	92.8	0.9727

### 2.3 检测平台的验证

本研究设置了阳性、阴性及空白对照,使用10 μg磁珠量(50 μL抗原量)、10 μg荧光微球量(5 μL二抗量)、10 μL样本量(样本1:20稀释)的体系,20 min内完成检测,检测结果分为明场和暗场图片,如图7所示。明场图片下可以观察到磁珠-抗原-抗体-二抗-荧光微球复合物的分散状态良好,暗场图片下则可以通过Image J软件获取平均荧光强度,再通过剂量-反应方程换算成浓度。阴性及空白样本的浓度分别为2.25 U/mL及1.69 U/mL。均低于9.97 U/mL,为阴性。阳性样本浓度为33.10 U/mL,高于9.97 U/mL,为阳性,符合预期。

**图7:**
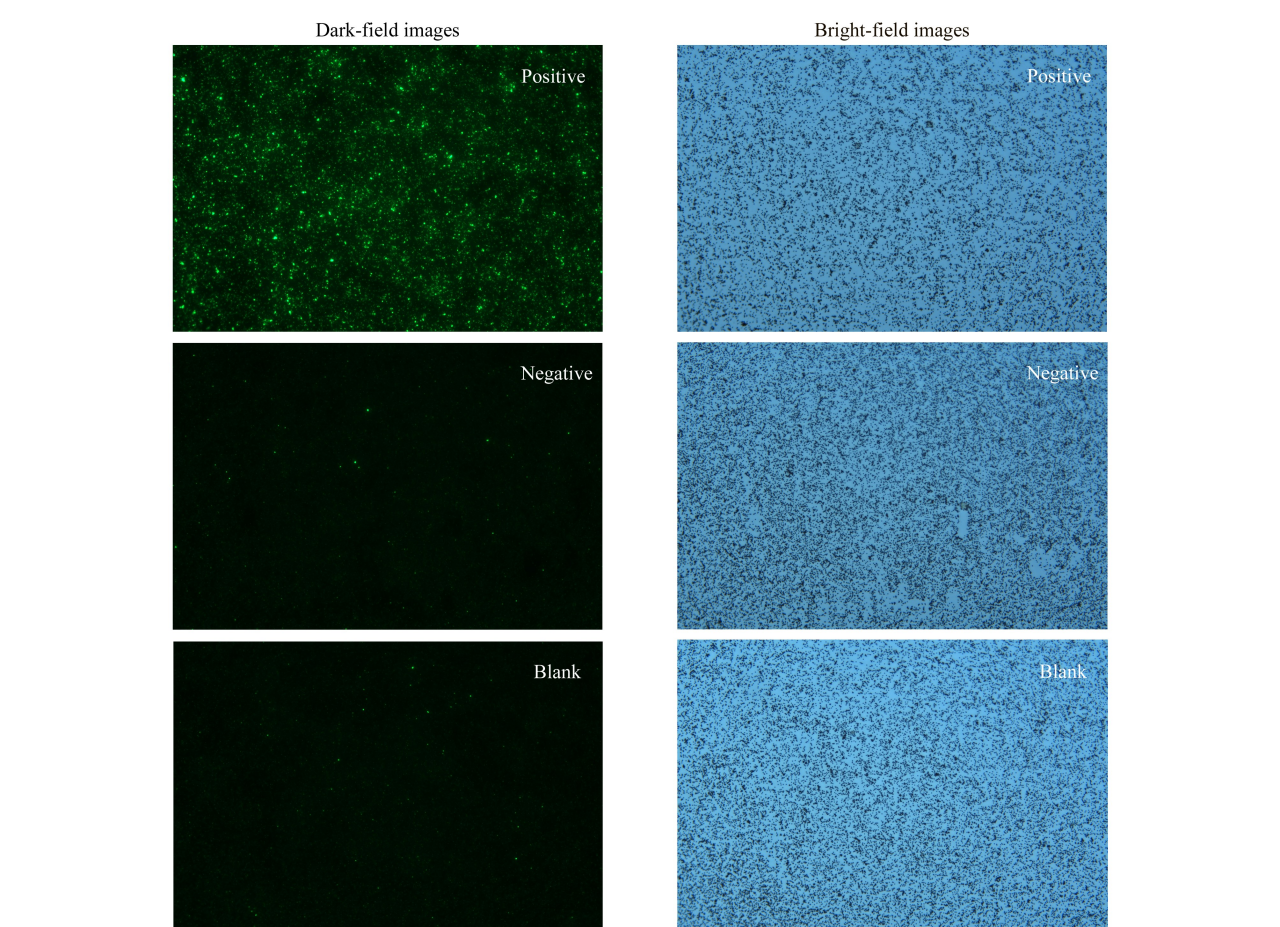
阳性、阴性及空白对照样本测试图(EB VCA IgG)

### 2.4 性能验证

各项目的剂量反应线性回归方程*R*^2^、线性范围和检出限见表4, *R*^2^均大于0.99, *P*值均小于0.05。

**表4:** 各项目的剂量反应线性关系和检出限

Item	Regression equation	*R* ^2^	Linear range/ (U/mL)	LOD/ (U/mL)
EB VCA IgG	*Y*=0.8532*X*+9.044	0.9984	1.92-200	1.92
EB VCA IgA	*Y*=1.150*X*+9.354	0.9958	2.79-200	2.79
EB NA1 IgG	*Y*=1.127*X*+7.172	0.9986	3.13-200	3.13
EB NA1 IgA	*Y*=1.191*X*+14.77	0.9952	1.53-200	1.53

*Y*: standard fluorescent signal value; *X*: standard concentration, U/mL; *R*^2^: correlation coefficient.

重复性试验结果如表5所示,4个项目的高低值样本的RSD均小于10%,但后续仍需进一步验证加速破坏条件下的精密度。

**表5:** 各项目重复性测试结果(*n*=10)

Sample No.	EB VCA IgG			EB NA1 IgG			EB VCA IgA			EB NA1 IgA	
	Average/(U/mL)	RSD/%		Average/(U/mL)	RSD/%		Average/(U/mL)	RSD/%		Average/(U/mL)	RSD/%
1	17.36	2.86		19.21	4.7		15.18	6.54		22.56	4.02
2	135.8	4.17		73.67	5.2		96.98	5.83		100.12	5.5

特异性测试结果显示,除与自身相同项目的阳性血清样本测试为阳性外,CMV、Toxo、MP、RV、VZV、EBV共6种病原体抗体阳性血清样本,微流控芯片测试反应均为阴性,表明本芯片的特异性较好。但后续仍需进一步验证芯片检测的抗体与其他疱疹病毒如单纯疱疹病毒1型及2型、卡波济肉瘤相关病毒、人类疱疹病毒6型及7型等是否有交叉反应。

### 2.5 与CLIA法的方法学比对

将本检测方法与CLIA检测方法的结果进行相关性分析。分别收集71例(EB VCA IgG)、50例(EB VCA IgA)、71例(EB NA1 IgG)及50例(EB NA1 IgA)临床上使用YHLO化学发光试剂盒验证为阳性的样本。同时收集50例(EB VCA IgG)、71例(EB VCA IgA)、50例(EB NA1 IgG)及71例(EB NA1 IgA)经过YHLO化学发光检测试剂盒测试为阴性的样本作为阴性对照。用本研究所建立的微流控磁免疫检测芯片进行检测,以本方法测得的浓度值为横坐标(*X*),以CLIA法测得的浓度值为纵坐标(*Y*),分析两种方法检测结果的相关性。EB VCA IgG、EB VCA IgA、EB NA1 IgG和EB NA1 IgA的线性回归方程分别为*Y*=0.9994*X*+0.09609(*R*^2^=0.9993)、*Y*=1.002*X*-0.02818 (*R*^2^=0.9979)、*Y*=1.002*X*+0.04766 (*R*^2^=0.9992)和*Y*=0.9953*X*+0.07087(*R*^2^=0.9994), *P*均<0.0001。各项目方法学比对的回归方程*R*^2^均大于0.99, *P*值均小于0.05,说明相关性较好。

利用SPSS 26.0软件对本检测方法与CLIA检测方法检测结果进行kappa一致性分析,两种方法阴阳性符合率均大于80%,各Kappa值均在0.8~1之间,各*P*值均小于0.05,可以认为两种方法的检测结果一致性较好,见表6。

**表6:** 各项目方法学比对及Kappa值

Item	Coincident rate		Kappa
	Positive	Negative	
EB VCA IgG	95.8% (68/71)	86.0% (43/50)	0.828
EB NA IgG	92.0% (46/50)	93.0% (66/71)	0.847
EB VCA IgA	93.0% (66/71)	92.0% (46/50)	0.847
EB NA IgA	90.0% (45/50)	91.6% (65/71)	0.813

利用Bland-Altman图对临床测试结果进行一致性分析,121例配对数据如图8所示,95%置信区间外的样本占比结果见表7。微流控芯片法与CLIA法测试结果相差的幅度在临床上可以接受,可以认为两种方法的测试结果具有较好的一致性。

**图8:**
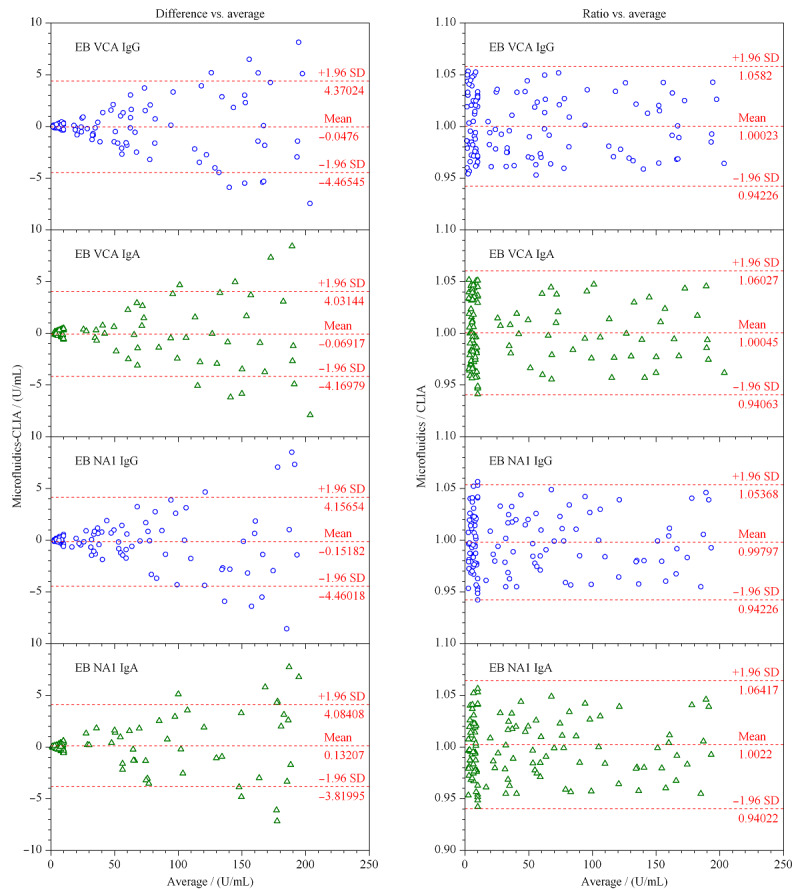
各项目的Bland-Altman绝对偏差和相对偏差统计图

**表7:** 各项目Bland-Altman图的95%置信区间外的样本占比

Item	Difference vs. average	Ratio vs. average
EB VCA IgG	8.3% (10/121)	0 (0/121)
EB VCA IgA	6.6% (8/121)	0 (0/121)
EB VCA IgG	7.4% (9/121)	1.7% (2/121)
EB VCA IgG	8.3% (10/121)	0 (0/121)

### 2.6 微流控试剂、仪器成本及优势分析

微流控试剂成本主要包括芯片原材料费用、压敏胶费用、磁珠费用、荧光微球费用、抗原及抗体费用。目前微流控芯片的材料主要有硅制材料、高聚合材料、陶瓷等,其中硅制材料及陶瓷材料虽然具有良好的加工性能,但这些材料加工时间较长,不适合量产。高聚合物材料同时兼具加工性能良好、成本低、加工时间短、加工工艺简单、易于产业化等优点^[33,34,35]^。故本实验选取高聚合物材料中的PMMA为芯片加工材料。若将本芯片投入产业化生产时,则需考虑利用模塑法代替激光切割工艺,这能进一步缩短芯片的加工时间。仪器成本主要包括离心模块费用、显微拍照模块费用、芯片固定模块费用、配套软件费用、顶针及磁吸模块费用。详见表8。

**表8:** 微流控芯片、试剂及仪器成本价目表

Material	Category	Cost/Yuan	Total/Yuan	Specification
Chip sheet	chip material	5-7	8.68-11.18	price from large production (10000+)
PSA	chip material	1-1.5		price from large production (10000+)
Magnetic beads	reagent material	0.2×4		price from commercial products
Fluorescent micro-particles	reagent material	0.08×4		price from commercial products
Antigen (EB VCA and EB NA1)	reagent material	(0.3+0.35)×2		0.3 for EB VCA and 0.35 for EB NA1, both from
				commercial products
Antibody (anti-human IgA and	reagent material	(0.03+0.1)×2		0.03 for anti-human IgG and 0.1 for anti-human
anti-human IgG)				IgA, both from commercial products
Centrifuge module	instrument material	2500-3000	6000-7500	price from commercial products
Microscope module	instrument material	2000-2500		price from commercial products
Fixation module	instrument material	300-400		price from commercial products
Valve control module	instrument material	300-400		price from commercial products
Magnet control module	instrument material	600-800		price from commercial products
Software		300-400		price from YHLO

相对于传统的ELISA方法,本方法的试剂成本及仪器成本比国内ELISA试剂成本(约2元人民币/测试)及仪器成本(约5000元人民币/台)高一些,但具有检测时间短、试剂消耗少、自动化程度高、污染少等优点。相对于CLIA方法,本方法的试剂成本(含芯片)与CLIA相差不大(国内CLIA试剂成本约10元人民币/测试),但本方法的仪器成本明显低于CLIA(国内CLIA仪器成本约150000元人民币/台)。同时还具有多项目联合检测、发光时间长、易于基层推广等优点。

## 3 结论

在本研究中,我们结合微流控技术及磁微粒免疫荧光分析技术建立的EB病毒标志物微流控检测平台经过性能验证及临床样本测试,检测验证与预期一致,临床样本检测结果显示与临床使用的CLIA法的测试结果具有较好的一致性,同时本平台还具有检测时间短、试剂消耗少、污染少、自动化程度高、易于基层推广等优点,可适用于各级医疗机构。
